# Effects of pica practice on oral bacteriome and mycobiome profiles among pregnant women: A comparative study

**DOI:** 10.1371/journal.pone.0328198

**Published:** 2026-05-08

**Authors:** Brenda A. Z. Abu, Lanxin Zhang, Robert Beblavy, Yan Wu, Xingyi Lu, Kevin Fiscella, Michael B. Sohn, Jin Xiao

**Affiliations:** 1 Wegmans School of Health and Nutrition, College of Health Sciences and Technology, Rochester Institute of Technology, Rochester, New York, United States of America; 2 Department of Molecular and Cell Biology, University of California‌‌, Berkeley, Oakland, California, United States of America; 3 Biostatistics and Computational Biology, University of Rochester, Medical Center, Rochester, New York, United States of America; 4 Eastman Institute for Oral Health, University of Rochester Medical Center, Rochester‌‌, New York, United States of America; 5 Department of Family Medicine, University of Rochester Medical Center, Rochester, New York, United States of America; King’s College Hospital NHS Foundation Trust, UNITED KINGDOM OF GREAT BRITAIN AND NORTHERN IRELAND

## Abstract

**Introduction:**

Pica, the excessive craving and consumption of non-food substances such as clay, and ice, is common among pregnant women but may pose risks for oral and systemic infections.

**Objective:**

Assessed the comparative effect of pica practice on the oral microbiome (bacteriome and mycobiome) profiles of pregnant women.

**Methods:**

A cross-sectional study was conducted in Upstate New York among pregnant women. Demographic, socioeconomic, pica practices (current and past), and oral hygiene practices were collected via questionnaires. The medical history of anemia was self-reported and verified using electronic records. A calibrated dentist assessed dental caries, periodontal status, and a comprehensive oral examination (plaque index, bleeding on probing). Oral samples (saliva and supragingival plaque) and pica samples were collected for the microbiome for Genomic DNA using I6S rRNA and ITS DNA sequencing and analyzed using linear regression with and without anemia as a covariate.

**Results:**

Of the 20 pregnant women in the study, 17 were minority women (75% non-white). The mean age of participants was 29 years, and 29 weeks of gestational age. Eight participants (40.0%) reported practicing pica, and six provided samples, namely ice (and popsicles), and chalk. *Streptococcus*, *Actinomyces*, and *Prevotella* dominated in both saliva and plaque samples, but the microbial compositions differed. Between the pica and the non-pica groups, two differentially abundant (DA) bacterial taxa were identified in saliva samples with and without anemia namely*Oribacterium sinus* (p < 0.05). In plaqueseven identical DA bacterial taxa including *Prevotella nigrescens* were seen *except for Leptotrichia goodfellowii*, which was unique to when anemia was controlled for *(*p < 0.05). Network analysis showed the co-occurrence of *Candida albicans and Lactobacillus* in the pica group.

**Conclusion:**

Pica practice was associated with specific oral taxa abundance change in saliva and supragingival plaque, reflecting distinct microbiome distributions. In the regression model, including anemia as a covariate had almost no impact on the overall DA results. These findings are preliminary, indicating that future large prospective cohort studies are warranted to thoroughly assess the impact of pica practice on oral flora.

## Introduction

Pica, the excessive craving and consumption of non-food substances such as clay, paint chips, and ice, is common among at-risk pregnant women. Pica is estimated to affect about 27.8% of pregnant and postpartum [1], yet the prevalence is estimated at up to 53.7% for US mothers aged 16–30 years with infants younger than 1 year of age and participating in the Special Nutrition Program on Women, Infant, and Children (WIC) [2]. In Rochester, New York, 46% of 158 pregnant teens reported pica behavior [3].

Pica practices are commonly associated with iron deficiency anemia (IDA) patients and during pregnancy [1]. Pica substances may pose a risk for oral and systemic infections. A few studies reported that geophagia (i.e., the pica for soil and gravel) and pagophagia (i.e., the pica for ice) caused tooth abrasions [4,5] while geophagia and amylophagia (i.e., the pica for uncooked starch like rice, and laundry starch) were associated with caries [4,6,7]. Studies reporting poor oral health outcomes among people who practiced pica also observed significant associations with low hemoglobin and serum ferritin levels [33,34].

Nutrition during the first 1000 days of life of a baby is critical for both oral and lifelong health outcomes [8]. Gestational iron deficiency within the first 1000 days is associated with pica behaviors in pregnant adolescents [3,9]. Both pica and IDA have severe consequences on birth outcomes [3,9] although the direct mechanisms are unclear. A systematic review of 78 studies indicated that the oral microflora of pregnant women might be influenced by oral and systemic conditions such as gestational diabetes [10]. Potential systemic effects during pregnancy include iron status [11] and dietary iron intake [12,13]. Thus, food access and security are critical [14] to improve oral and overall health. Food assistance programs such as WIC [15] close the food access gap for many households.

The maternal microbiome during pregnancy may have adverse effects on neonatal and infant health [16]. Thus, prenatal oral health plays a pivotal role in the health of the mother and unborn babies. For instance, a systematic review of observational studies confirms that pregnant women who have periodontal diseases are at an increased risk for adverse birth outcomes [17].

Although knowledge regarding the impact of pica practices during pregnancy on the overall health of mothers and babies is evolving, the role of pica in the oral microbiome is yet to be studied. We assessed the comparative effect of pica practice on oral bacteriome and mycobiome profiles of pregnant women. We hypothesize that pica may affect the oral microbiome through two routes: locally, directly introduced microorganisms from pica substances to the oral cavity, and systemically through infections. Findings are exploratory to generate hypotheses for further studies to guide early warning of oral disease or risks, antenatal health screening during pregnancy, and education program development.

## Materials and methods

### Study design, population, and sample

The Institutional Review Board of the Rochester Institute of Technology, New York, reviewed the research protocol of this cross-sectional study and approved it (HSRO # 05040921). Participants were recruited from Upstate New York through social media, the Prenatal Dental Clinic of URMC, and other community programs. All participants were informed of the study objectives and protocol before they gave informed consent to participate n the study..

For this exploratory comparative study, 10–40 pregnant women were expected [18].

### Inclusion and exclusion criteria

The inclusion criteria were: 1) female, older than 18 years of age; 2) self-reported pregnancy; 3) willing to participate in this study and complete two follow-up appointments; 4) provided verbal or written informed consent. Signed informed consent was saved on either Qualtrics or paper and locked in a secure location. The exclusion criteria were: 1) inability to provide informed consent; 2) received oral and/or systemic antibacterial or antifungal therapy within 90 days of the baseline study visit; 3) requiring premedication before dental treatment; 4) presence of orofacial deformity or tumor, e.g., cleft lip/palate; and 5) self-reported severe systemic diseases that could severely impair the immune system such as HIV and cancer.

### Data collection tools and procedures

All assessments were conducted between May 1, 2022, and August 30, 2023. The authors obtained access to information that could identify individual participants during data collection, which was used to verify health history from electronic records. Participants underwent baseline visits for interviews with questionnaires and a second visit for oral examination, and oral and pica sample collection. The questionnaire encompassed demographics collected using a validated questionnaire. Medical backgrounds and medications were self-reported and verified using electronic records, encompassing: 1) physician-diagnosed systemic diseases (Y/N), anemia; 2) supplements taken; 3) pica characteristics such as onset of pica, length of pica practice, and experienced pica in previous pregnancy [19]; 4) Food security was assessed using the six-item United States Department of Agriculture (USDA) [14]. Socioeconomic backgrounds and oral hygiene practices were also assessed using a questionnaire.

Oral examination was conducted by one of the two trained and calibrated dentists on pregnant women in a dedicated examination room using standard dental examination equipment, materials, and supplies. Caries status was assessed using Decayed, Missing, and Filled Teeth (DMFT) and the International Caries Detection and Assessment System (ICDAS) criteria [20]. Dental plaque was evaluated using the Plaque Index (PI) following Löe’s criteria, with scores ranging from 0 to 3 for each of the four gingival areas of the tooth. Bleeding on probing (BOP) was measured to assess gingival inflammation. Periodontal tissue assessment involves probing the bottom of the clinical pocket or sulcus with a periodontal probe. Interproximal sites for all existing teeth, excluding third molars, were evaluated from both buccal and lingual sides. Participants reported orofacial pain using the Numeric Rating Scale (NRS) score (0–10, with 10 indicating unbearable pain). Inter- and intra-examiner agreement for evaluated criteria was calculated using Kappa statistics and exceeded 90% following calibration.

Participants provided whole non-stimulated saliva samples by spitting into a sterilized 50 ml centrifuge tube. Before their study visit, participants were instructed not to eat, drink, or brush their teeth for 2 hours before the oral sample collection. Approximately 2 ml of saliva was collected from each subject. Supragingival plaque from the whole dentition was collected using a sterilized periodontal scaler. The plaque sample was suspended in 1 ml of 0.9% sodium chloride solution in a sterilized Eppendorf tube. Participants practicing pica brought their pica substance samples for microbial analysis. Pica samples were received and transferred to a sterile tube to reduce contamination. Samples were transported to the lab within 2 hours and stored in the −80 °C freezer.

#### Sample preparation and oral microbiome analysis.

Oral samples (saliva, plaque, and pica substances) were stored on ice and transferred within 2 hours to the laboratory at the Eastman Institute for Oral Health, University of Rochester, New York. Saliva and plaque samples were gently vortexed and sonicated (10 seconds sonication, 30 seconds rest on ice, repeated three times) to disaggregate the plaque before plating. About 250 mg of each pica substance was soaked in 1 ml of 0.9% sodium chloride solution.

DNA extraction and 16S rRNA and ITS DNA sequencing were carried out at Diversigen Inc. (New Brighton, Minnesota). Genomic microbial DNA from salivary and plaque samples (100ul saliva and 200ul plaque suspension) was extracted using Quick-DNA™ Fecal/Soil Microbe Miniprep Kit (ZymoResearch, Irvine, CA) [21–24]. The bacterial 16S rRNA V1-V3 hypervariable region and fungal ITS region were amplified and sequenced on an Illumina MiSeq [24,25]. The sequencing library was prepared using an innovative library preparation process in which PCR reactions were performed in real-time PCR machines to control cycles and, therefore, limit PCR chimera formation. The final PCR products were quantified with qPCR fluorescence readings and pooled together based on equal molarity. The final pooled library was cleaned up with the Select-a-Size DNA Clean & Concentrator™ (Zymo Research, Irvine, CA), then quantified with TapeStation® (Agilent Technologies, Santa Clara, CA) and Qubit® (Thermo Fisher Scientific, Waltham, WA). The ZymoBIOMICS® Microbial Community Standard (Zymo Research, Irvine, CA) was used as a positive control for each DNA extraction performed. The ZymoBIOMICS® Microbial Community DNA Standard (Zymo Research, Irvine, CA) was used as a positive control for each targeted library preparation. Negative controls (i.e., blank extraction control, blank library preparation control) were included to assess the level of bioburden carried by the wet-lab process. The final library was sequenced on Illumina® MiSeq™ with a V3 reagent kit (600 cycles). The sequencing was performed with a 10% PhiX spike-in.

### Data analysis

#### Demographic and clinical characteristics.

Descriptive statistics were calculated for demographic and clinical characteristics. Food security was analyzed using the USDA guidelines [14]. Chi-square tests were used for race, ethnicity, employment, education, marital status, medical background, and food security. Independent sample t-tests were used to assess mean differences in Age, Gestational week, PI, DMFT, and DMFS. Mann-Whitney U test for non-normally distributed variables, ICDAS, and BOP.

#### Microbiome and statistical analysis.

QIIME 2 [26] was used to quantify the composition and diversity of each community. Alpha diversity was computed using the Shannon index, and significance was assessed using the t-test. For beta diversity, the Bray-Curtis distance was used. Principal Coordinate Analysis (PCoA) was used to visualize the results of beta diversity, and permutational multivariate analysis of variance (PERMANOVA) was used to test statistical significance. The relative abundance at different levels was plotted to visualize the differences in microbial compositions between pica and non-pica participants.

To identify differentially abundant (DA) taxa between non-pica and pica groups, we employed a linear regression model with arcsine-transformed data, with the pica practice as the main predictor and decayed teeth and anemia as covariates. The regressions were repeated without anemia as a covariate to assess the effect of anemia on the DA analysis. Because of a limited sample size, taxa appearing in fewer than 20% of samples were filtered out in DA analysis to reduce false positives.

For both plaque and saliva samples, co-occurrence analyses were conducted using the Ising model, separately for pica practice and racial group. The optimal penalty was selected using the extended Bayesian information criterion. Data is stored in the National Institutes of Health (NIH) Sequence Read Archive (SRA) database. The identification number is PRJNA1185228.

## Results

### Background characteristics of participants‌‌

Recruited women (n = 20) were between the ages of 22 and 35 years in the 2nd or 3rd trimester of pregnancy (Table 1). About half (55.0%) were of African American heritage, 50.0% had a college education or higher, and the rest had high school (20.0%) or associate degrees (25.0%). About 80.0% were employed and mostly unmarried (80.0%).

**Table 1:** Sociodemographic characteristics of the participants (N = 20).

Variable	Categories	Total (n = 20) % (n)	Non-pica (n = 12) % (n)	Pica (n = 8) % (n)	*p-*value
*Demographic-socioeconomic*					
Age (year, Mean± SD)		29.65 ± 6.0	30.92 ± 6.7	27.8 ± 5.0	0.27
Gestational Week (Mean± SD)		25.55 ± 8.8	25.42 ± 9.7	25.75 ± 8.7	0.47
Race	African American	55.0 (11)	41.7 (5)	75.0 (6)	0.14
	Others	45.0 (9)	58.3 (7)	25.0 (2)	
Ethnicity	Hispanic	20.0 (4)	16.6 (2)	25.0 (2)	0.65
	Non-Hispanic	80.0 (16)	83.3 (10)	75.0 (6)	
Employment (Yes)		80.0 (16)	83.3 (10)	75.0 (6)	0.65
Education	≤ High school	20.0 (4)	16.7 (2)	25.0 (2)	0.67
	Associate degree	25.0 (5)	25.0 (3)	25.0 (2)	
	≥ College	50.0 (10)	58.3 (7)	37.5 (3)	
Marital Status	Married	20.0 (4)	16.7 (2)	25.0 (2)	0.65
	Non-married	80.0 (16)	13.3 (10)	75.0 (6)	
*Medical condition, pregnancy symptoms, and medication history*					
Anemia		25.0 (5)	8.3 (1)	50.0 (4)	0.03*
Perinatal supplement usage		89.5 (17)	90.9 (10)	87.5 (7)	0.81
Reported pregnancy symptoms	Vomiting	50.0 (10)	49.8 (6)	25.0 (4)	–
	Nausea	40.0 (8)	49.8 (6)	50.0 (8)	–
	Change in appetite	45.0 (9)	41.5 (5)	50.0 (4)	–
	Diarrhea	20.0 (4)	16.7 (2)	25.0 (2)	–
	Constipation	45.0 (9)	41.5 (5)	50.0 (4)	–
*Pica practices and characteristics*					
Pica type	Ice (from participants	35.0 (7)	–	87.5 (7)	–
	Ice (curated from the market)	(7)	–	–	–
	Popsicle (from participants)	5.0 (1)	–	8.4 (1)	–
				
	Chalk (from participants	5.0 (1)	–	1 (8.4)	–
	Chalk (curated from the market)	(1)	–	–	–
	Clay (curated from the market)	(2)	–	–	–
#Poly pica		10.0 (2)	0 (0.0)	16.6 (2)	–
Onset of pica	Pre-pregnancy	20.0 (4)	0 (0.0)	33.2 (4)	–
	During pregnancy	20.0 (4)	0 (0.0)	33.2 (4)	
Length of pica practice	≤ 1 year	20.0 (4)	0 (0.0)	33.2 (4)	–
	>1 year	20.0 (4)	0 (0.0)	33.2 (4)	
Experienced pica in previous pregnancy		15.0 (3)	0 (0.0)	25.0 (3)	
Food security status	Food secure	80.0 (16)	66.4 (8)	49.8 (6)	0.667
	Food insecure	20.0 (4)	16.7 (2)	16.6 (2)	
Participating in food assistance program	WIC	65.0 (13)	49.8 (6)	41.6 (7)	–
	SNAP	15.0 (3)	8.3 (1)	8.4 (2)	–
*Hygiene, Oral condition, and outcome parameters*					
Frequency of Brushing	Twice/daily	80.0 (16)	83.3 (10)	75.0 (6)	0.65
	≤ Once/daily	20.0 (4)	16.7 (2)	25.0 (2)	
Plaque index^(Mean± SD)^		0.65 [0.25, 0.89]	0.61 [0.19, 1.05]	0.68 [0.30, 0.89]	0.62
DMFT score ^(Mean± SD)^		5.55 ± 4.4	3.67 ± 3.4	8.38 ± 4.6	0.03*
DMFS score ^§^		9.85 ± 9.1	5.58 ± 4.9	16.25 ± 11.1	0.02*
ICDAS score ^§^		2.2 [2.0, 2.8]	2.11 [2.0, 2.6]	2.35 [2.0, 3.1]	0.47
BOP ^§^		0.5 [0.0, 3.00]	0.0 [0.0, 3.0]	1.0 [0.0, 8.0]	0.62

DMFT: decayed, missing, filled teeth; DMFS: decayed, missing, filled surface; BOP: Bleeding on Probing and ICDAS: International Caries Detection and Assessment System; WIC: the Special Supplemental Nutrition Program for Women, Infants, and Children; SNAP: Supplemental Nutrition Assistance Program: § Indicates median [interquartile range]; and * indicates p < 0.05; Chi-square tests were used for race, ethnicity, employment, education, marital status, medical background, and food security. Independent sample t-tests were used to assess mean differences in Age, Gestational week, Plaque Index, DMFT, and DMFS. Mann-Whitney U test for comparison of the medians and interquartile range differences in ICDAS and BOP. # Poly pica is the pica for more than one substance.

From the medical conditions’ history, 25.0% reported a history of anemia, with a significant proportion (50% pica versus 8.3% non-pica; p = 0.03) of them practicing pica. A high (89.5%) perinatal supplement usage was observed, with no differences (p = 0.81) observed between pica and non-pica groups. No significant differences were observed in the pregnancy symptoms between the pica and non-pica groups, and vomiting was higher in the pica group (100.0%) compared to the non-pica group (49.8%).

Overall, 40% practiced pica. No significant differences were observed between the pica and non-pica groups. Poly pica was observed in two, i.e., 25.0% of pica participants. The onset of pica during pre-pregnancy and pregnancy was equally distributed. Overall, food insecurity was low (10.0%), although slightly higher for the pica group (25.0%) compared with the pica group (16.7%). Most of the participants participated in WIC (65.0%), with the pica group having a higher participation (87.5%).

For oral hygiene and oral health outcome parameters, 80.0% of the women reported brushing their teeth twice a day, and the rest brushed once a day. No differences were observed for the plaque index, ICDAS score, and BOP for pica and non-pica groups. The DMFT score (p = 0.03) and DMFS score (p = 0.02) significantly differed between pica and non-pica groups, showing that individuals who practice pica have more decayed, missing, and filled teeth and surfaces.

### Profile and diversities of oral bacteriome and mycobiome among pica and non-pica users

#### Oral bacteriome and mycobiome profiles.

**Fig 1:**
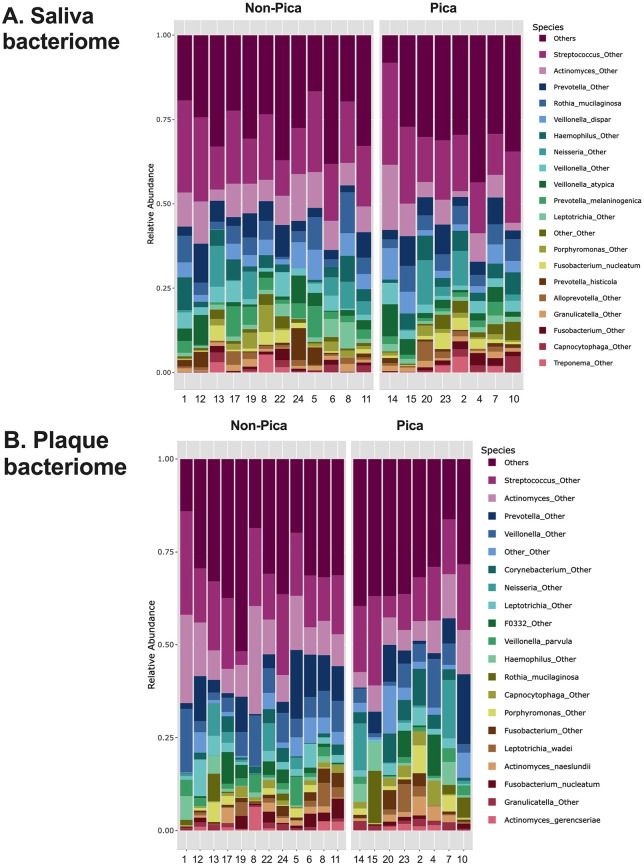
Profile of oral bacteriome among pica and non-pica participants (n = 20). **Legend:** The relative abundance of the microbial composition of saliva (A) and plaque (B) for each participant, categorized based on the treatment group (pica or non-pica), is shown. The 20 most abundant species in the merged salivary samples are presented.

**Fig 2:**
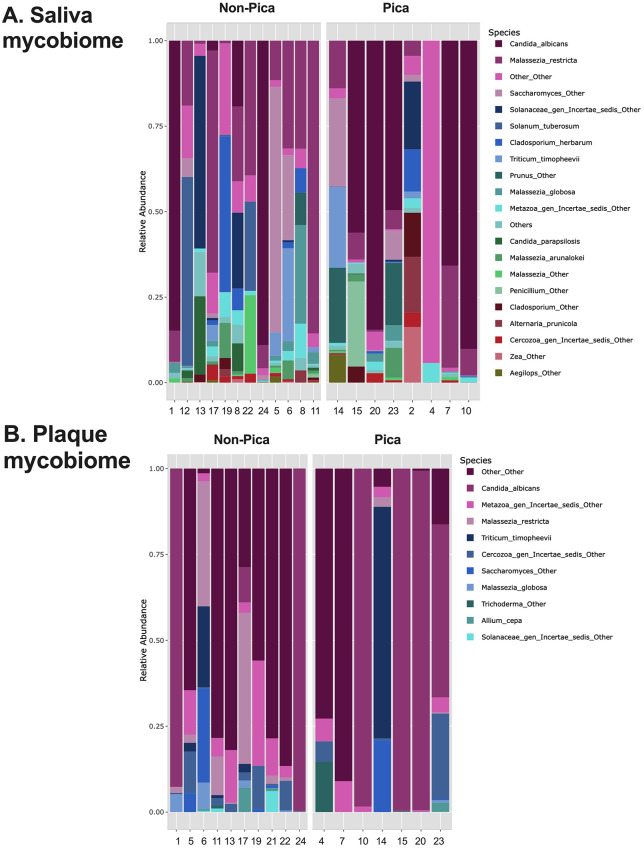
Profile of oral mycobiome among pica and non-pica participants (n = 20). **Legend:** The fungal composition of saliva (A) and plaque (B) for each subject, categorized based on the treatment group (pica or non-pica), is shown. The 20 most abundant species in the merged salivary samples are presented.

Figs 1A and 1B show the 20 most abundant species in plaque and saliva samples, respectively. Although species from *Streptococcus*, *Actinomyces*, and *Prevotella* dominate in both saliva and plaque, the species with lower relative abundance vary, including species from *Haemophilus*, *Veillonella*, *Leptotrichia*, and others. It is also evident that the microbiome composition varies greatly among the subjects. Figs 2A and 2B demonstrate that the fungal composition varies greatly among subjects, indicating that participants exhibit different mycobiomes.

#### Comparison of the diversity of salivary and plaque bacteriome and mycobiome between pica and non-pica users.

We compared the oral microbial diversity between individuals with pica and non-pica. For saliva samples, alpha diversity, as measured by the Shannon index, showed no statistically significant differences between the pica and non-pica groups (bacteriome: p = 0.76; mycobiome: p = 0.79), indicating similar levels of microbial diversity (S1A and S2A Figs). Beta diversity analysis, which assesses the composition of microbial communities, also showed no significant differences between the groups in saliva samples for both bacteriome (p = 0.86) and mycobiome (p = 0.65), with overlapping clusters in the PCoA plots (S1B and S2B Figs).

Similarly, plaque samples demonstrated no significant differences in microbial diversity between the Pica and Non-Pica groups. The Shannon index for alpha diversity showed no statistical differences for both bacteriome (p = 0.81) and mycobiome (p = 0.29) analyses, indicating comparable microbial richness and evenness in plaque samples across the two groups (S1C and S2C Figs). Beta diversity analysis of plaque samples also showed overlapping clusters for pica and non-pica individuals (bacteriome: p = 0.79; mycobiome: p = 0.18), suggesting no distinct variation in microbial composition based on pica practice (S1D and S2D Figs). Overall, these findings indicate that pica practices do not appear to be significantly associated with microbial diversity in either saliva or plaque samples, according to both alpha and beta diversity measures‌‌.

#### Differentially abundant bacterial taxa between the pica and non-pica groups.

**Fig 3:**
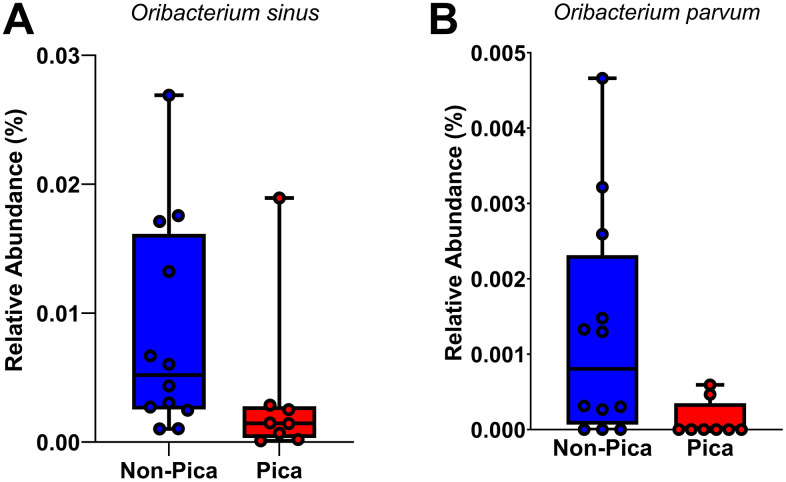
Differentially abundant bacteria in saliva between pica and non-pica participants (n = 20). **Legend:** Linear regression model with arcsine-transformed data, with the pica practice as the main predictor and decayed teeth and anemia as a covariate. Two (2) taxa, namely *Oribacterium sinus* and *Oribacterium parvum*, were significant*.* These are illustrated for pica and non-pica participants.

**Fig 4:**
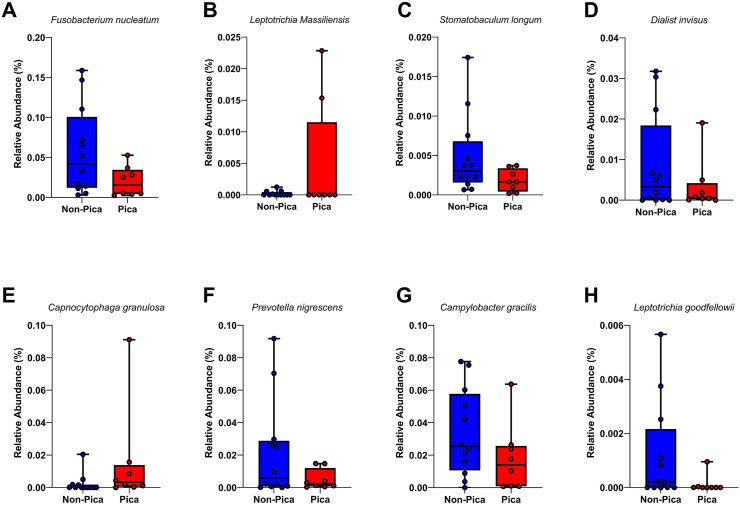
Differentially abundant bacteria in plaque between pica and non-pica participants (n = 30). **Legend:** Linear regression model with arcsine-transformed data, with the pica practice as the main predictor and decayed teeth and anemia as covariates. Eight (8) taxa reached a raw p-value < 0.05. namely*. Fusobacterium nucleate, Leptotrichia Massilliensis, Prevotella nigrescens, Stomatobaculum longum, Dialist invisus, Capnocytophaga granulosa, Campylobacter gracilis,* and *Leptotrichia goodfellowii***.** Graphs show that for pica and non-pica participants.

In the 16S rRNA dataset, 580 initial taxa were reduced to 111 in saliva samples and 96 in plaque samples after filtering out taxa appearing fewer than two times in both the pica and non-pica groups. For the ITS dataset, 258 initial taxa were reduced to 28 in saliva samples and 13 in plaque samples. From the logistic regression using pica as a predictor and controlling for anemia and decayed teeth, for both saliva and plaque. In the regression model, including anemia as a covariate had almost no impact on the overall DA results. For instance, for saliva, 2 identical taxa, i.e., *Oribacterium sinus* and *Oribacterium parvum,* were seen to be significant whether anemia was controlled for or not (Fig 3). For plaque samples, when anemia was controlled for, 8 taxa reached a raw p-value < 0.05. Significant taxa included *Fusobacterium nucleate*, *Leptotrichia Massilliensis, Prevotella nigrescens, Stomatobaculum longum, Dialist invisus, Capnocytophaga granulosa, Campylobacter gracilis, and Leptotrichia goodfellowii (*Fig 4). When anemia was present in the regression model, 7 identical taxa reached a raw p-value < 0.05. The only additional taxon identified in plaque when anemia was included was *Leptotrichia goodfellowii*. Analysis showing, linear regression model with the pica practice as the main predictor and decayed teeth as a covariate and anemia was not controlled is added as supplementary material for saliva (S5 Fig) and plaque (S6 Fig).No differentially abundant taxa were identified in the ITS saliva or plaque samples.

No differentially abundant fungal taxa were identified between the pica and non-pica users.

### Network analysis of microorganisms in plaque and saliva samples

The results from the network analysis for both the plaque and saliva samples are displayed in an undirected graph. The nodes represent taxa, and the edges represent nonzero partial correlations between taxa. Larger nodes indicate that a taxon was present in a larger proportion of samples. The color of each node represents the group membership. The edges for each plot are colored to indicate the direction of partial correlation. The dark grey edges indicate a positive partial correlation, while the light brown edges indicate a negative one. Thicker edges indicate a stronger partial correlation between pairs of taxa.

S3A Fig shows the co-occurrence of taxa in saliva samples from racial groups. Not many nodes appeared in both groups (purple nodes), indicating a difference in co-occurrence between the two racial groups. *M. globosa* occurred in both black and white participants and had a negative co-occurrence with *C. matruchotri* in black and *P. abscessus* in white participants. S3B Fig shows the microbial co-occurrence in plaque samples for both racial groups. No common nodes appeared in both groups, and more taxa co-occurred in the black group, indicating a significant difference in the co-occurrence of taxa between the racial groups.

S3C Fig shows the co-occurrence of taxa in saliva samples from participants with and without pica practice. Four out of 48 nodes appeared in both groups, indicating a difference in the co-occurrence pattern between the pica and non-pica groups. The pica group had a lesser number of co-occurring taxa compared to the non-pica group. Notably, the co-occurrence of *C. albicans* and *Lactobacillus* was detected in the pica group. S3D Fig shows the microbial co-occurrence in plaque samples from participants with and without pica practice. Only two nodes appeared in both groups, and one of them, *A. geminatus* co-occurred with *C. albicans* in the pica group and *J. ignava* in the non-pica group.

### Profile of pica substances bacteriome

Overall, eight participants (40.0%) reported practicing pica, and six of them provided samples. The reported substances included ice (80.0%) and chalk (20%) (Table 1). For procured (n = 6) samples 80.0%, of ice samples were procured from commercial avenues. Chalk (10.0%) and clay (10.0%) procured from commercial avenues yielded no bacterium, although the chalk sample procured from the participant showed predominant *Pelomonas Aquatica.* Similarly, ice procured from participants showed varying levels of *Brevundimonas* and *Pseudomonas* compared to the predominant *Pelomonas Aquatica* in the ice samples procured from commercial venues*.* Only 10.0% of ice samples from participants had a high proportion of *Escherichia Shigella.* While 60.0% (out of the 80.0%) samples procured from commercial avenues contained *Escherichia Shigella,* as shown in supplemental material (S4 Fig).

## Discussion

In this comparative study, the objective was to assess the effects of pica practices on the oral microbiome profile of pregnant women. Of the 20 pregnant women, half were of African American heritage, 40% practiced pica (chalk and ice), and 60% did not practice pica. Pica was significantly associated with a medical history of anemia. Pica participants had *C. albicans* in both saliva and plaque, with a co-occurrence with *Lactobacillus* in saliva samples. Pica samples obtained from participants contained *Brevundimonas* and *Pseudomonas* compared to the predominant *Pelomonas Aquatica and Escherichia Shigella* in pica (ice) procured from commercial avenues. Food insecurity was 25.0% among the pica group compared with 16.5% among the non-pica group. Common reported pregnancy symptoms were nausea and vomiting during pregnancy. Five taxa in saliva samples, including *Lactobacillaceae Lactobacillus,* and four [4] taxa in plaque were differentially abundant. All four [4] taxa from plaque were more abundant in the pica group compared to the non-pica group. From network analysis, a co-occurrence of *Candida albicans and Lactobacillus* was observed among women who practice pica, unlike the non-pica group.

Of the 30% who practiced pica, 75% were African Americans, and 50% had a history of anemia. These findings align with previous findings that reported that pica practices were common among Hispanics and African Americans [3]. Recent studies have shown a strong association with nutrient deficiencies, such as IDA, with pica [27]. Although food access and insecurity among these populations may pose additional risks from pica [14]. A study showed an association between pica, with iron deficiency, and food insecurity among Hispanic pregnant women [27]. In another study of 158 pregnant teenagers in upstate New York, 75% were African Americans. That study reported 45% with a pica for ice [3]. Socioeconomically disadvantaged children are more likely to have higher *Candida* carriage [28]. The presence of pica practices may further increase their risk for candida carriage and caries risk.

In this comparative study of saliva and plaque samples, we observed *C. albicans* in the pica group but not the non-pica groups. For the pica participants, the co-occurrence of *C. albicans* and *Lactobacillus* was observed in the profiles of saliva samples. These findings need further verification, given that *C. albicans,* a cariogenic microorganism [29], is modulated by changes in the oral pH [30]. In vitro studies, iron facilitates *C. albican* growth, abundance, and virulence [31,32]. In human studies, systemic iron status, denoted by anemia status, especially for IDA, is strongly associated with pica [33] and [33–36]. *Lactobacillus,* on the other hand, is a contributor to dental caries, although its specific role and the mechanisms in human caries development are underresearched. In in vitro studies, *Lactobacill*i have poor biofilm-forming ability compared to *Streptococcus mutans,* a major *pathogen for human dental caries.* Other evolving evidence states that *Lactobacillus,* in the presence of sugar, may have a detrimental effect on microorganisms close to it [37], although these findings are preliminary. Since *C. albicans* is an independent risk factor for dental caries, further investigations exploring the occurrence of *C. albicans* and its co-occurrence with *Lactobacillus* on pica samples need to be further examined with a larger sample size. The co-occurrences preliminarily show correlation, not functional interaction. The biological relevance is not clearly established.

Pica samples, i.e., chalk and clay procured from commercial avenues, yielded no bacterium, although the chalk sample procured from the participants showed predominant *Pelomonas aquatica. Shigella* was observed in ice samples procured from both participants and commercial avenues. Our findings align with other studies of common pica samples observed by different strains of microbes, such as clay and soil [38]. The highest microorganism profiles observed in those samples were mostly *staphylococcus.* Other organisms isolated from their samples procured from commercial venues in Ghana were *Klebsiella Escherichia, Enterobacter, Shigella* [38]. Although the direct effects of *Shigella* and *Pelomonas aquatica* on oral health are unclear, both have overall health implications when consumed. For example, *Shigella* triggers foodborne illness that may cause gastrointestinal symptoms [39]. The predominant *Pelomonas aquatica* from the profiles has previously been isolated from industrial and hemodialysis water [40]. These conclusions reflect relative rather than absolute abundance, and future studies should include qPCR or culture-based assays.

In this sample, anemia did not have a major effect on the taxa identified in the DA analysis for saliva, but did to a smaller extent in the plaque samples. Two identical DA taxa were reported in saliva when anemia was controlled for, and when it was not, in a linear regression. For plaque samples, seven identical taxa were observed, except for *Leptotrichia goodfellowii*, which was uniquely differentially abundant when anemia was controlled for. Anemia may affect immune functions and can independently alter oral microbiota. A few studies have shown that anemia, especially IDA, is associated with caries [41,42]. A significantly higher detection rate of salivary *C. albicans* was observed in the anemic group (80.0% versus 26.7% in the non-anemic, p = 0.035) [33]. Both high and low iron act as an environmental signal in multiple signaling pathways that alter cell wall architecture in *C. albicans*, thereby affecting its survival upon exposure to antifungals and host immune response [31]. As *Leptotrichia goodfellowii,* which ferments carbohydrates to produce lactic acid that may trigger tooth decay [43], the relationship with anemia needs further testing.

Our finding that *Capnocytophaga granulosa and Leptotrichia massiliensis,* were more abundant profiles in pica individuals is novel with potential oral health implications. For instance, *Capnocytophaga spp. and Capnocytophaga granulosa* have been implicated in periodontal disease [44]. Similarly, *Leptotrichia massiliensis* is involved in carbohydrate fermentation, leading to tooth decay [43].

Lastly, *Comamonas* spp. are associated with environmental bioremediation that is considered non-pathogenic, but may also reside in water or the gut microbiome [45]. Pica practice and pregnancy symptoms, such as vomiting, may introduce *Comamonas* to the mouth. As these findings are exploratory, they warrant further investigation with a larger sample size and diverse pica patients (such as amylophagia or geophagia) using qPCR or culture-based assays to understand their contributions to oral microbiome dynamics. In our sample, we observed *Comamonas and Pelomonas.* There is a limited and unclear biological significance to these identified taxa. For instance, *Comamonas,* which are nonpathogenic, and their presence may indicate water contamination. Also, *Pelomonas* aquatica isolated from pica samples is not typically an oral pathogen. We recommend further analysis of these pica samples and corresponding saliva to assess the oral and overall health implications of these taxa that potentially are introduced to the oral region through various pica samples.

From the DMFT and DMFS scores, we observed that the pregnant women who practiced pica had more decayed, missing, and filled teeth and surfaces, even though oral hygiene practices such as brushing their teeth were not different between groups. The DMFT and DMFS scores are strongly associated with caries [46]. These oral health indicators confirm that pica practices may be associated with oral health outcomes compared to non-pica counterparts. The comparative differences in their bacteriome and mycobiome profiles may explain the observed differences between pica and non-pica groups, although these findings are exploratory.

### Limitations

The results of this comparative study should be interpreted with consideration of the study’s limitations, which may limit generalizability. First, the sample size was relatively small, although there was variability in socio-demographic variables. To obtain significant results with the measured effect sizes (0.79 for saliva and 1.02 for plaque), the sample sizes of 65 per group for saliva and 40 per group for plaque are required to achieve 80% power at a family-wise error rate of 0.05 using the Bonferroni-Holm correction [47] assuming similar numbers of taxa in these samples. Thus, the present ﬁndings should be replicated and followed up in larger and more diverse pregnant women as well as child-mother dyads to identify effects on pregnancy outcomes, and onward relationships between the mother and child’s microbiome, and other oral health indices.

In our study, we used I6S rRNA and ITS DNA sequencing. Both methods are prone to primer biases, leading to underrepresentation, quantification, or failure to detect. This limitation was observed for some pica samples. Subject to funding, future studies may use complementary methods such as whole-genome or metagenomic sequencing for a fuller picture to refine our findings. Another limitation of this study is that 16S rRNA and ITS sequencing provided relative abundance rather than absolute microbial load. Complementary approaches, such as qPCR or culture-based quantification for total microbial load, were not performed, which could be done in future studies.

Finally, these preliminary data collected reflected current pica practices. The length of pica and the type of pica, which may help determinethe pica practice characteristics that increase the risk of oral health and dental disease conditions, were not collected.

## Conclusions

Pica practice was associated with specific oral taxa abundance in saliva and supragingival plaque. In the pica and the non-pica groups, five differentially abundant (DA) bacterial taxa were identified in saliva samples, including *Lactobacillaceae Lactobacillus*, and four DA bacterial taxa in plaque. Additionally, the co-occurrence of both *C. albicans* and *Lactobacillus* among women who practice pica could further provide insight into the potential relationship between pica practice and cariogenic microorganism profiles. For the other microbiomes common among pregnant women who practiced pica, direct impacts on oral health may not be apparent, though indirect impacts through other infections and consequent immune status changes are possible. Future prospective cohort studies with a bigger sample size are warranted to comprehensively assess the impact of pica practice on oral flora. These conclusions reflect relative rather than absolute abundance, and future studies should consider including qPCR or culture-based assays.

### Knowledge transfer statement

Craving and consumption of non-food‌‌ or non-nutritive substances is called pica. Pica practices were reported among 40% of pregnant women. Pica substances, i.e., ice and chalk, and saliva and plaque of the pica group showed different microbial compositions. Women in the pica group were more likely to be anemic and have decayed, missing, and filled teeth and surfaces. The findings highlighted the potential benefit of assessing pica practices of pregnant women during oral health risk screening.

## Supporting information

S1 Fig Alpha and Beta Diversity of saliva and plaque bacteriome among pica and non-pica users (n = 20). Legend: For saliva (A) and plaque (C) samples, alpha diversity bacteriome was not statistically different for pica status using the Shannon index. Beta diversity shows no distinct variation in microbial composition based on pica practice for saliva (B) and plaque (D) samples.
(DOCX)

S2 Fig Alpha and Beta Diversity of saliva and plaque mycobiome among pica and non-pica users (n = 20). **Legend:** For saliva (A) and plaque (C) samples, alpha diversity of the mycobiome was not statistically different for pica status using the Shannon index. Beta diversity shows no distinct variation in microbial composition based on pica practice for saliva (B) and plaque (D) samples.
(DOCX)

S3 Fig Bacteriome and Mycobiome Network Analysis for saliva and plaque (n = 20). **Legend:** Co-occurrence between taxa stratified by racial groups for saliva (A) and plaque (B) samples and by pica status for saliva (C) and plaque (D) samples. Only those taxa found to have a significant co-occurrence with at least one other taxon are displayed in the plots.
(DOCX)

S4 Fig Relative abundance of bacteria in pica samples (n = 10). **Legend**: The figure shows the relative abundance of bacteria for 10 pica samples; four (4) were ice samples (S41, S43, S44, S44), 1 (S45) popsicle,and 1 (S49) chalk sample were procured from participants. Four (4) ice samples were directly procured from commercial avenues (S51, S54, S56, S58).
(DOCX)

S5 Fig Differentially abundant bacteria in saliva (n = 20). **Note:** Linear regression model with arcsine-transformed data, with the pica practice as the main predictor and decayed teeth as a covariate. Anemia was not controlled for. Two (2) taxa, namely *Oribacterium sinus* and *Oribacterium parvum*, were significant. These are illustrated for pica and non-pica participants.
(DOCX)

S6 Fig Differentially abundant bacteria in plaque (n = 20). **Note:** Linear regression model with arcsine-transformed data, with the pica practice as the main predictor and decayed teeth as covariates. Anemia was not controlled for. Seven (7) taxa reached a raw p-value < 0.05. namely*. Fusobacterium nucleate, Leptotrichia Massilliensis, Prevotella nigrescens, Stomatobaculum longum, Dialist invisus, Capnocytophaga granulosa, and Campylobacter gracilis*. Graphs show that for pica and non-pica participants.
(DOCX)
