# Supplementary material for: Effects of pica practice on oral bacteriome and mycobiome profiles among pregnant women: A comparative study
Source: PLoS One. 2026 May 8;21(5):e0328198. doi: 10.1371/journal.pone.0328198 (PMC13155548; doi:10.1371/journal.pone.0328198)
Supplement: S1 Fig — Legend: For saliva (A) and plaque (C) samples, alpha diversity bacteriome was not statistically different for pica status using the Shannon index. Beta diversity shows no distinct variation in microbial composition based on pica practice for saliva (B) and plaque (D) samples. (DOCX) [file pone.0328198.s001.docx]

**Effects of pica practice on oral bacteriome and mycobiome profiles among pregnant women: a comparative study:** Brenda A.Z. Abu^1^, Lanxin Zhang^2^, Robert Beblavy^3^, Yan Wu^4^, Kevin Fiscella^5^, Xingyi Lu^4^, Micheal B. Sohn^3^, Jin Xiao^4^.

**
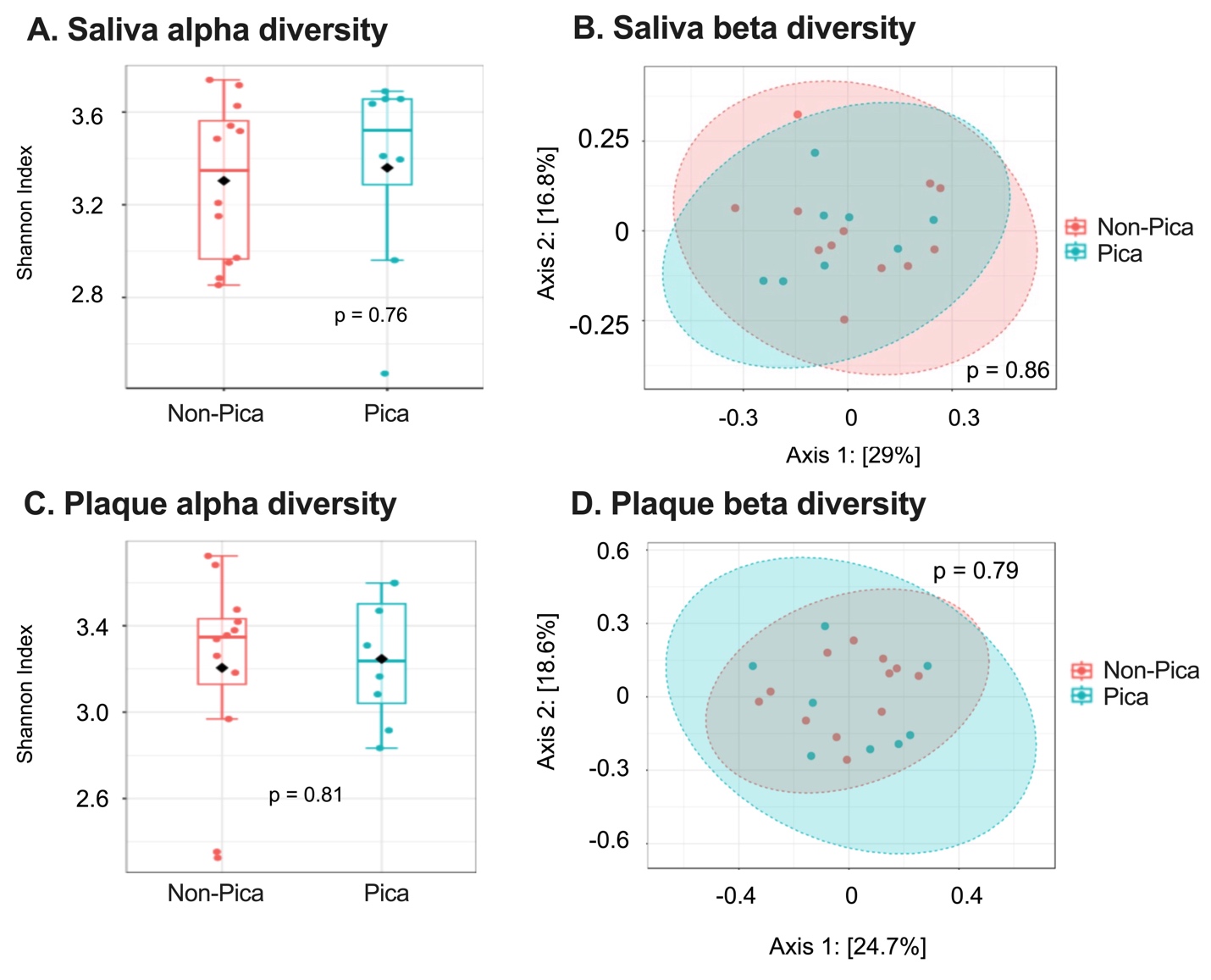
**

**S Fig 1. Alpha and Beta Diversity of saliva and plaque bacteriome among pica and non-pica users**

**Legend:** For saliva (A) and plaque (C) samples, alpha diversity bacteriome was not statistically different for pica status using the Shannon index. Beta diversity shows no distinct variation in microbial composition based on pica practice for saliva (B) and plaque (D) samples.
