# Supplementary material for: Effects of pica practice on oral bacteriome and mycobiome profiles among pregnant women: A comparative study
Source: PLoS One. 2026 May 8;21(5):e0328198. doi: 10.1371/journal.pone.0328198 (PMC13155548; doi:10.1371/journal.pone.0328198)
Supplement: S3 Fig — Legend: Co-occurrence between taxa stratified by racial groups for saliva (A) and plaque (B) samples and by pica status for saliva (C) and plaque (D) samples. Only those taxa found to have a significant co-occurrence with at least one other taxon are displayed in the plots. (DOCX) [file pone.0328198.s003.docx]

**Effects of pica practice on oral bacteriome and mycobiome profiles among pregnant women: a comparative study:** Brenda A.Z. Abu^1^, Lanxin Zhang^2^, Robert Beblavy^3^, Yan Wu^4^, Kevin Fiscella^5^, Xingyi Lu^4^, Micheal B. Sohn^3^, Jin Xiao^4^.


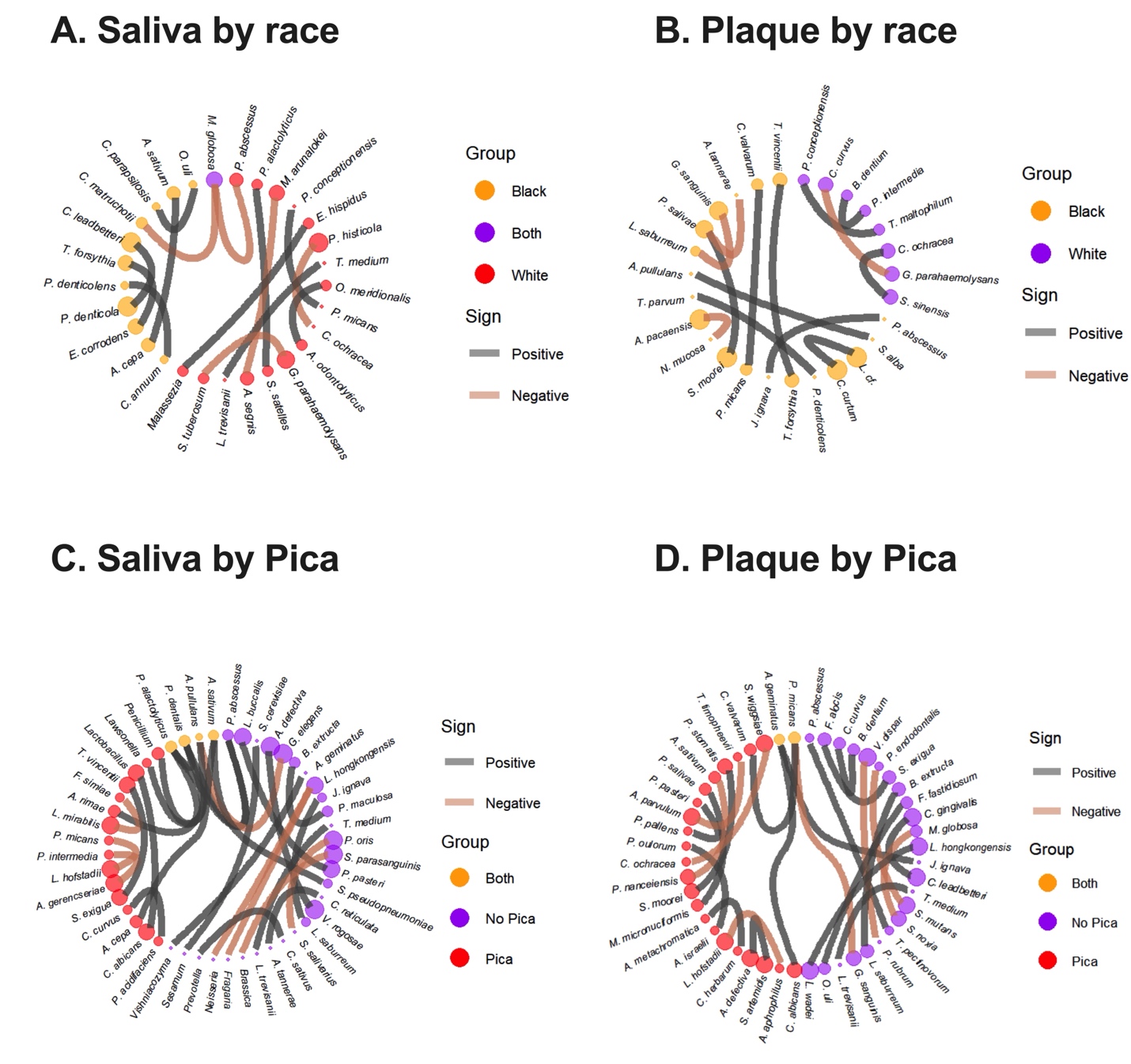


**S Fig 3. Bacteriome and Mycobiome Network Analysis for saliva and plaque**

**Legend:** Co-occurrence between taxa stratified by racial groups for saliva (A) and plaque (B) samples and by pica status for saliva (C) and plaque (D) samples. Only those taxa found to have a significant co-occurrence with at least one other taxon are displayed in the plots.
