# Supplementary material for: Effects of pica practice on oral bacteriome and mycobiome profiles among pregnant women: A comparative study
Source: PLoS One. 2026 May 8;21(5):e0328198. doi: 10.1371/journal.pone.0328198 (PMC13155548; doi:10.1371/journal.pone.0328198)
Supplement: S4 Fig — Legend: The figure shows the relative abundance of bacteria for 10 pica samples; four (4) were ice samples (S41, S43, S44, S44), 1 (S45) popsicle,and 1 (S49) chalk sample were procured from participants. Four (4) ice samples were directly procured from commercial avenues (S51, S54, S56, S58). (DOCX) [file pone.0328198.s004.docx]

**Effects of pica practice on oral bacteriome and mycobiome profiles among pregnant women: a comparative study:** Brenda A.Z. Abu^1^, Lanxin Zhang^2^, Robert Beblavy^3^, Yan Wu^4^, Kevin Fiscella^5^, Xingyi Lu^4^, Micheal B. Sohn^3^, Jin Xiao^4^.

**
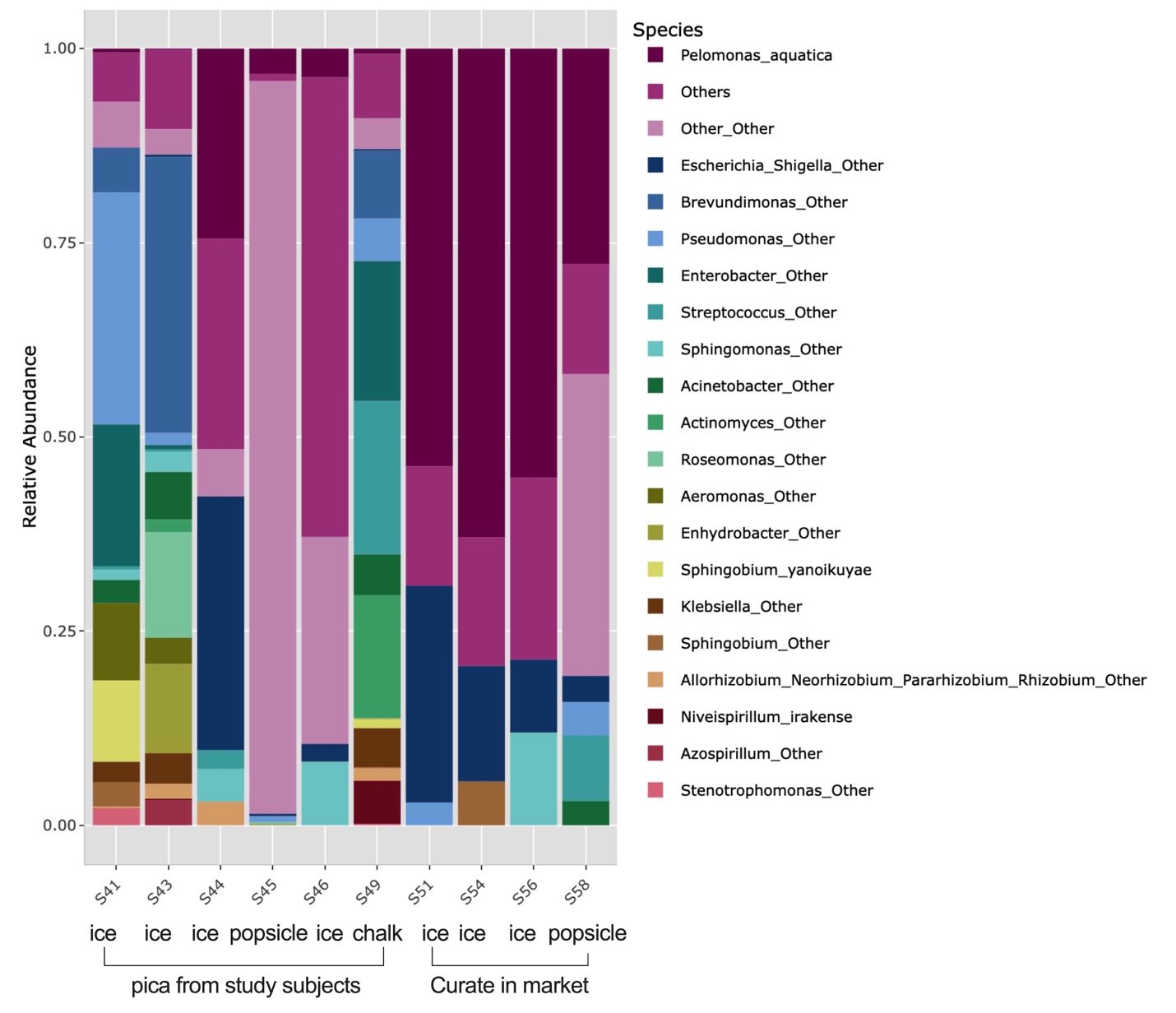
**

**S Fig 4: Relative abundance of bacteria in pica samples**

**Legend: The figure shows the relative abundance of bacteria for 10 pica samples; 4 were ice samples (S41, S43, S44, S44) procured from participants, 1 (S45) popsicle was procured from a participant, and four ice samples were procured from commercial avenues (S51, S54, S56, S58).**
