# Supplementary material for: Effects of pica practice on oral bacteriome and mycobiome profiles among pregnant women: A comparative study
Source: PLoS One. 2026 May 8;21(5):e0328198. doi: 10.1371/journal.pone.0328198 (PMC13155548; doi:10.1371/journal.pone.0328198)
Supplement: S5 Fig — Note: Linear regression model with arcsine-transformed data, with the pica practice as the main predictor and decayed teeth as a covariate. Anemia was not controlled for. Two (2) taxa, namely Oribacterium sinus and Oribacterium parvum, were significant. These are illustrated for pica and non-pica participants. (DOCX) [file pone.0328198.s005.docx]

**Effects of pica practice on oral bacteriome and mycobiome profiles among pregnant women: a comparative study:** Brenda A.Z. Abu^1^, Lanxin Zhang^2^, Robert Beblavy^3^, Yan Wu^4^, Kevin Fiscella^5^, Xingyi Lu^4^, Micheal B. Sohn^3^, Jin Xiao^4^.


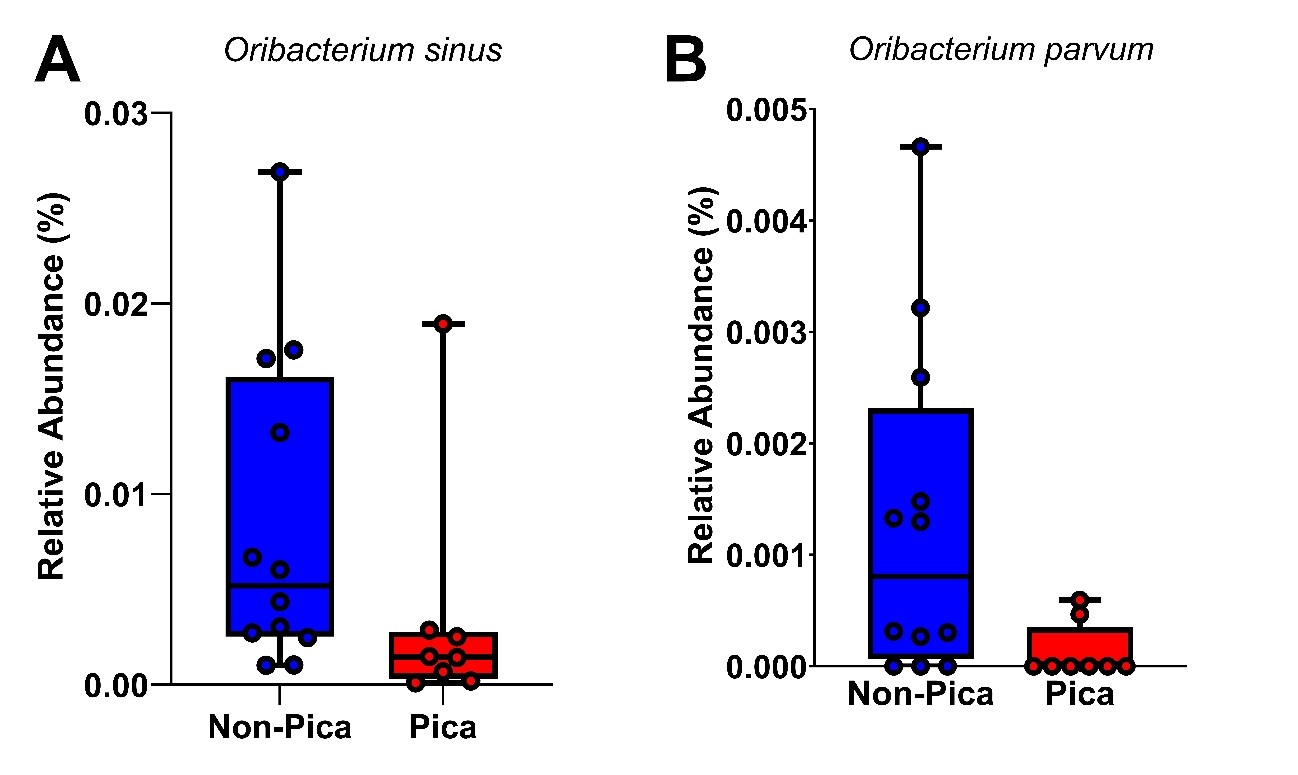


**Fig S5. Differentially abundant bacteria in saliva.**

**Note: Linear regression model with arcsine-transformed data, with the pica practice as the main predictor and decayed teeth as a covariate. Anemia was not controlled for.** Two (2) taxa, namely *Oribacterium sinus* and *Oribacterium parvum*, were significant*.* These are illustrated for pica and non-pica participants.
