# Supplementary material for: Effects of pica practice on oral bacteriome and mycobiome profiles among pregnant women: A comparative study
Source: PLoS One. 2026 May 8;21(5):e0328198. doi: 10.1371/journal.pone.0328198 (PMC13155548; doi:10.1371/journal.pone.0328198)
Supplement: S6 Fig — Note: Linear regression model with arcsine-transformed data, with the pica practice as the main predictor and decayed teeth as covariates. Anemia was not controlled for. Seven (7) taxa reached a raw p-value < 0.05. namely. Fusobacterium nucleate, Leptotrichia Massilliensis, Prevotella nigrescens, Stomatobaculum longum, Dialist invisus, Capnocytophaga granulosa, and Campylobacter gracilis. Graphs show that for pica and non-pica participants. (DOCX) [file pone.0328198.s006.docx]

**Effects of pica practice on oral bacteriome and mycobiome profiles among pregnant women: a comparative study:** Brenda A.Z. Abu^1^, Lanxin Zhang^2^, Robert Beblavy^3^, Yan Wu^4^, Kevin Fiscella^5^, Xingyi Lu^4^, Micheal B. Sohn^3^, Jin Xiao^4^.

**
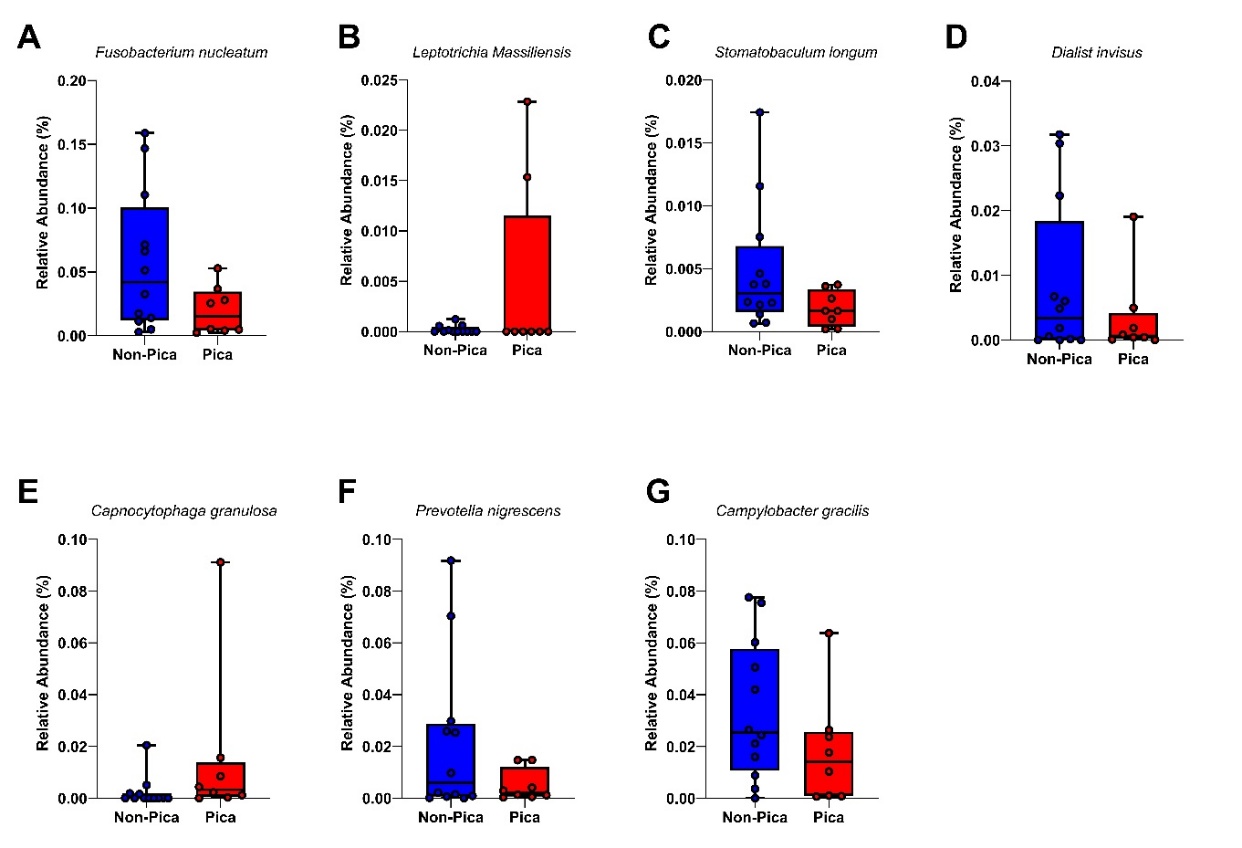
**

**Fig S6. Differentially abundant bacteria in plaque**

**Note: Linear regression model with arcsine-transformed data, with the pica practice as the main predictor and decayed teeth as covariates. Anemia was not controlled for. Seven (7)** taxa reached a raw p-value < 0.05. namely*. Fusobacterium nucleate, Leptotrichia Massilliensis, Prevotella nigrescens, Stomatobaculum longum, Dialist invisus, Capnocytophaga granulosa, and Campylobacter gracilis***.** Graphs show that for pica and non-pica participants.
